# Detection of increased pyruvate dehydrogenase flux in the human heart during adenosine stress test using hyperpolarized [1-^13^C]pyruvate cardiovascular magnetic resonance imaging

**DOI:** 10.1186/s12968-022-00860-6

**Published:** 2022-06-06

**Authors:** Steen Hylgaard Joergensen, Esben Soevsoe S. Hansen, Nikolaj Bøgh, Lotte Bonde Bertelsen, Peter Bisgaard Staehr, Rolf F. Schulte, Craig Malloy, Henrik Wiggers, Christoffer Laustsen

**Affiliations:** 1 Department of Clinical Medicine, Aarhus University Hospital, The MR-Research Centre, Palle Juul Jensens Boulevard 99, 8200 Aarhus N, Denmark; 2 Department of Clinical Medicine, Department of Cardiology, Aarhus University Hospital, Hjoerring, Denmark; 3 Department of Cardiology, North Denmark Regional Hospital, Bispensgade 37, 9800 Hjoerring, Denmark; 4 GE Healthcare, Munich, Germany; 5 Advanced Imaging Research Center, University of Texas Southwestern Medical Center, Dallas, USA

**Keywords:** Cardiac metabolism, Stress test, Metabolic imaging, Perfusion

## Abstract

**Background:**

Hyperpolarized (HP) [1-^13^C]pyruvate cardiovascular magnetic resonance (CMR) imaging can visualize the uptake and intracellular conversion of [1-^13^C]pyruvate to either [1-^13^C]lactate or ^13^C-bicarbonate depending on the prevailing metabolic state. The aim of the present study was to combine an adenosine stress test with HP [1-^13^C]pyruvate CMR to detect cardiac metabolism in the healthy human heart at rest and during moderate stress.

**Methods:**

A prospective descriptive study was performed between October 2019 and August 2020. Healthy human subjects underwent cine CMR and HP [1-^13^C]pyruvate CMR at rest and during adenosine stress. HP [1-^13^C]pyruvate CMR images were acquired at the mid-left-ventricle (LV) level. Semi-quantitative assessment of first-pass myocardial [1-^13^C]pyruvate perfusion and metabolism were assessed. Paired t-tests were used to compare mean values at rest and during stress.

**Results:**

Six healthy subjects (two female), age 29 ± 7 years were studied and no adverse reactions occurred. Myocardial [1-^13^C]pyruvate perfusion was significantly increased during stress with a reduction in time-to-peak from 6.2 ± 2.8 to 2.7 ± 1.3 s, p = 0.02. This higher perfusion was accompanied by an overall increased myocardial uptake and metabolism. The conversion rate constant (*k*_PL_) for lactate increased from 11 ± 9 *10^–3^ to 20 ± 10 * 10^–3^ s^−1^, p = 0.04. The pyruvate oxidation rate (*k*_PB_) increased from 4 ± 4 *10^–3^ to 12 ± 7 *10^–3^ s^−1^, p = 0.008. This increase in carbohydrate metabolism was positively correlated with heart rate (R^2^ = 0.44, p = 0.02).

**Conclusions:**

Adenosine stress testing combined with HP [1-^13^C]pyruvate CMR is feasible and well-tolerated in healthy subjects. We observed an increased pyruvate oxidation during cardiac stress. The present study is an important step in the translation of HP [1-^13^C]pyruvate CMR into clinical cardiac imaging.

*Trial registration* EUDRACT, 2018-003533-15. Registered 4th of December 2018, https://www.clinicaltrialsregister.eu/ctr-search/search?query=2018-003533-15

## Background

Despite advanced imaging techniques, the selection of patients for revascularization remains a highly contested issue in both chronic coronary artery disease (CAD) and chronic heart failure (HF) [1–3]. The dissolution dynamic nuclear polarization technique (dDNP) and hyperpolarized (HP) [1-^13^C]pyruvate cardiovascular magnetic resonance (CMR) have emerged as promising methods for real-time, non-invasive imaging of cardiac metabolism [4, 5]. Hyperpolarized (HP) [1-^13^C]pyruvate CMR has the potential to monitor focal lactate accumulation in the myocardium and thus directly image an important metabolic feature often attributed to myocardial ischemia. Viable myocardium is detected by monitoring pyruvate dehydrogenase (PDH) flux [6–8]. Recently, Apps et al. published an initial report on two patients with CAD following myocardial infarction, showing a reduced bicarbonate signal in non-viable myocardial segments and a preserved signal in viable [9]. These encouraging results call for evaluation of HP [1-^13^C]pyruvate CMR in conjunction with conventional CMR adenosine stress testing to assess inducible myocardial ischemia. The primary purpose of this study was to investigate the feasibility and tolerability of HP [1-^13^C]pyruvate CMR in combination with adenosine vasodilator stress. A secondary purpose was to study the physiological changes in metabolism and myocardial perfusion in the healthy human heart during an adenosine stress.

## Methods

### Study population

We prospectively included healthy subjects aged over 18 years with no history of heart disease, no significant medical history, normal electrocardiogram (ECG) and normal echocardiography with a left ventricular (LV) ejection fraction (LVEF) > 50%. The study was conducted according to the Helsinki principles and approved by the National Committee on Health Research Ethics (2018-003533-15) and by the Danish Medicines Agency (2,019,123,690). Written informed consent was obtained from all participants before enrollment. Participants were recruited through local advertisement. The study was performed at the Department of Cardiology and at the MR-Research Centre, Aarhus University Hospital, Aarhus, Denmark between October 2019 and August 2020.

### Design

**Fig. 1:**
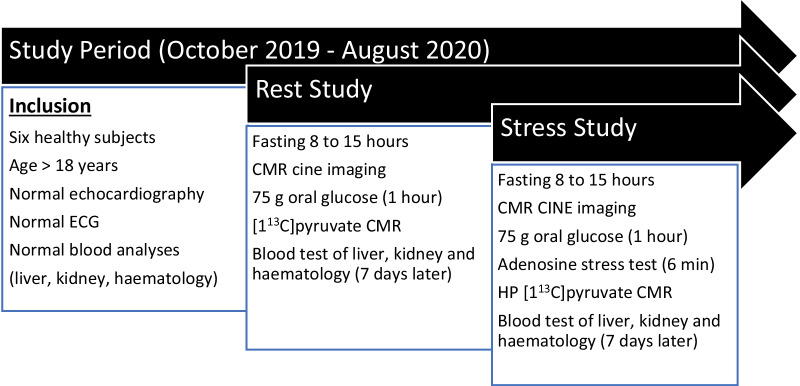
Outline of study design. The duration of each study visit was < 2 h. CMR cine imaging typically lasted 45 min and metabolic imaging lasted 5 min at rest and 6 min during adenosine stress test. The mean time interval between rest and stress study was 5 ± 3 months

A resting study and a stress study were performed on two separate days (Fig. 1). Participants fasted for 8 to15 h prior to study. Standard blood counts and metabolic profiles before and 7 days after each study day was done. Echocardiography was performed at the first visit (VIVID e9, General Electric Healthcare, Chicago, Illinois, USA) to assess systolic and diastolic function. ECG was recorded to confirm sinus rhythm and exclude abnormalities. Participants ingested 75 g of oral glucose in 200 ml water to maximize PDH flux [6]. Cine CMR imaging was performed with the subjects in the supine position. HP [1-^13^C]pyruvate CMR was performed one hour after glucose ingestion. Before adenosine stress, participants were instructed to refrain from caffeine for 24 h prior to the test. For the stress study, continuous adenosine infusion was initiated 1 h after glucose ingestion at a rate of 140 mcg/kg/min through an 18G venous cannula in the left arm using an infusion pump (Alaris™ VP Plus Guardrails™ Denmark) [10]. Three minutes later, hyperpolarized [1-^13^C]pyruvate was injected through an 18G venous cannula in the right antecubital vein using a power injector (Medrad®, Bayer Healthcare, Denmark) and hyperpolarized CMR was undertaken. A three-slice cine‐LV function assessed LV function during the last minute of adenosine infusion.

### Hyperpolarized CMR imaging

**Fig. 2:**
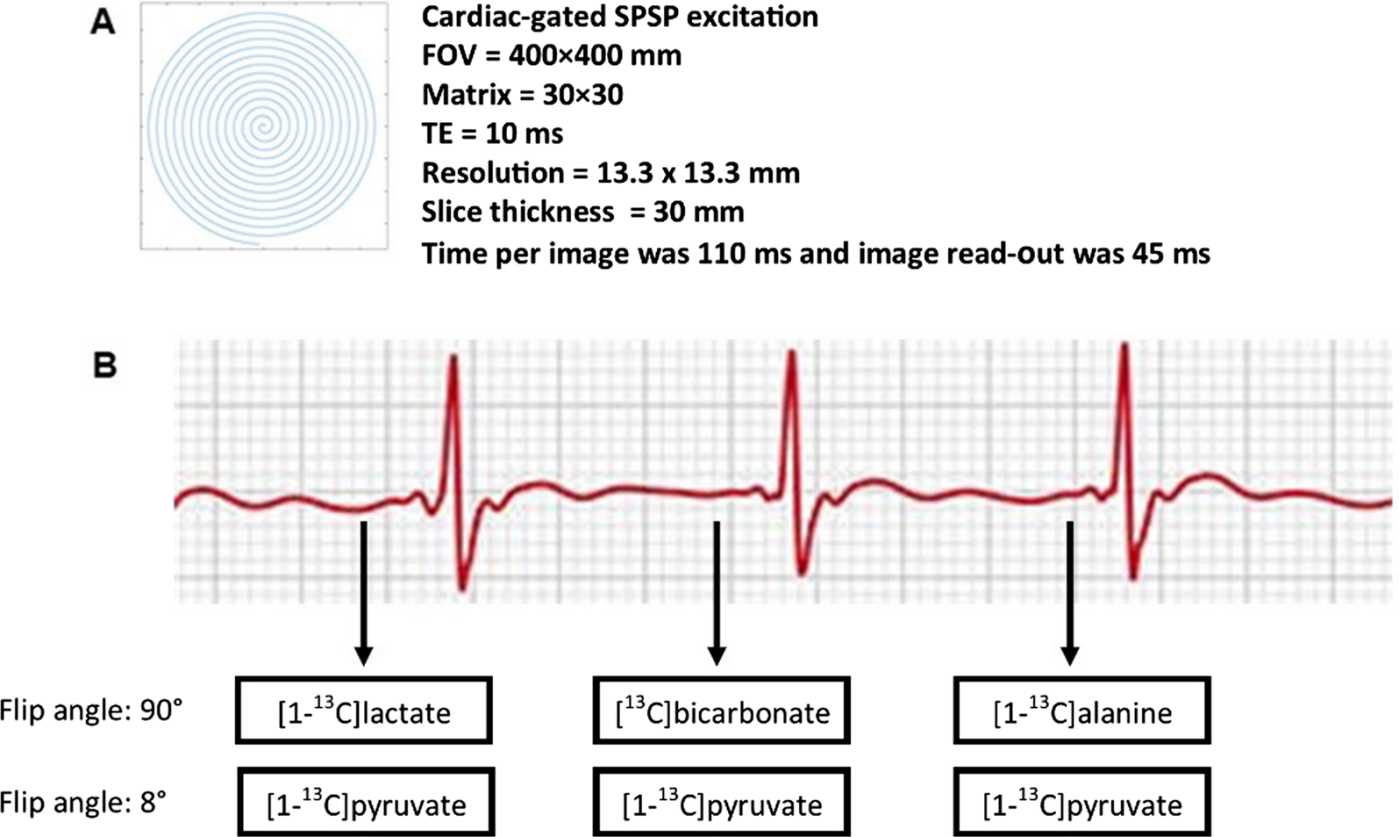
**A** Hyperpolarized imaging acquisition parameters. **B** Sequence diagram. Hyperpolarized data was acquired in diastole. Images of pyruvate were acquired in every heart beat and images of each of the metabolites were obtained for every third heart beat in the order: lactate, bicarbonate and alanine

The sterile [1-^13^C]pyruvate was polarised in a clinical SPINlab™ DNP polarizer (General Electric Healthcare) and release criteria were as previously described [14]. Proton images were acquired using an eight‐channel cardiac array receiver coil (General Electric Healthcare) or the built-in body coil. The same setup was used for each individual at both study dates. Balanced steady-state free precession (bSSFP) was used for cine LV function assessment. The sequence parameters were: TE 2.4 ms, TR 5.1 ms, flip angle 55°, acquisition matrix 200 × 160, FOV 400 × 400 mm^2^, in-plane resolution 2 × 2.5 mm^2^, slice thickness 8 mm, recon matrix 512 × 512 and cardiac phases 30. HP [1‐^13^C]pyruvate CMR was undertaken with a transmit/receive Helmholtz loop-pair ^13^C coil (PulseTeq Limited, UK) or a transmit clamshell coil with a 16-channel array receive coil (Rapid Biomedical GmbH, Rimpar, Germany). The multiple radiofrequency (RF) coil data were combined using a singular value decomposition (SVD method) [11]. Transmit gain calibration was performed to adjust the RF power levels to the desired flip angles for each subject and ^13^C transmit power was calculated with a Bloch-Siegert method on a [^13^C]-bicarbonate phantom positioned in the coil sensitivity area and close to the imaging plane above the heart [12]. Transmit gain was measured to vary ~ 0.2 dB across subjects. The imaging frequency for the HP ^13^C-imaging was calculated from the proton frequency obtained in the individual heart [13]. Iterative ^1^H B0 maps was obtained prior to ^13^C imaging to ensure a local B0 field of ± 15 Hz across the heart. The ^1^H centre frequency in the heart was then used to calculate the ^13^C frequencies as the same shim was used. HP imaging was acquired in diastole using a cardiac-gated spectral-spatial (SPSP) excitation with spiral read-out acquisition: TE = 10 ms, FOV = 400 × 400 mm, matrix = 30 × 30, real in-plane pixel size = 13.3 × 13.3 mm and one short axis slice with 30 mm slice thickness [14, 15]. Excitation pulses increase the decay of HP signal. We used a spiral acquisition scheme with an 8° flip angle for the precursor pyruvate to preserve signal and 90° for downstream metabolites lactate, bicarbonate and alanine to maximize metabolite signal. During each R-R interval, we used an excite-read in the diastolic phase for pyruvate and one other metabolite. Time per image was 110 ms and image read-out was 45 ms. We obtained 240 images. Thus, we obtained 120 pyruvate images and 40 images of each metabolite with a time resolution of three heart beats. (Fig. 2).

### Data analysis and statistics

Reconstruction of the ^13^C data and analysis of the DICOM images were done using Segment version 3.1 R8215 (http://segment.heiberg.se). ROIs outlining the myocardium was firstly drawn on the corresponding heart phase on the cine images and thereafter transferred to the ^13^C images. Here the ROIs were reduced to cover 5% less in endo and epi direction to reduce signal outside the myocardium. All images were tracked manually through the course of the acquisition to minimize motion due to breathing.

The metabolite signal was analysed at the mid-LV level as a mean signal from the myocardium. The area under the curve (AUC) from the peak pyruvate signal and ten time frames forward for [1‐^13^C]pyruvate and each of the metabolites were used to calculate the total myocardial signal using MATLAB (The Mathworks, Natick, Massachusetts, USA) [16, 17]. The exchange rate constants for the conversion of [1^13^C]pyruvate to [^13^C]lactate (*k*_PL_), [^13^C]bicarbonate (*k*_PB_) and [^13^C]alanine (*k*_PA_) were measured as previously described [18, 19].

Statistical analyses were made in GraphPad Prism Windows, (version 8.0.0, GraphPad Software, San Diego, California, USA). Normality was assessed using the Shapiro-Wilks test. Results are presented as mean ± standard deviation (SD). Paired t-tests were used to compare mean values from the two study days. One-tailed *p*-values were used for time to peak and first order moment pyruvate perfusion. Otherwise two-tailed *p*-values were used. A *p*-value < 0.05 was considered statistically significant.

## Results

### Population characteristics

Six healthy subjects, 29 ± 7 years were studied. Echocardiography showed normal diastolic function and CMR showed normal systolic function. Mean body mass index (BMI), mean glycated haemoglobin (HbA1c), mean fasting glucose were similar and within normal range on both study days (Table 1). Time from oral glucose to hyperpolarized [1-^13^C]pyruvate injection was 1 h ± 5 min. During the adenosine stress test, the heart rate (HR) increased from 65 ± 13 to 108 ± 11 bpm (*p* < 0.001) and the rate-pressure product (mmHg*bpm/10^3^) increased from 8.2 ± 1.1 to 12.3 ± 1.2 (*p* = 0.03) (Table 2).

**Table 1:** Population characteristics (mean ± SD)

Study population (n = 6)	Rest study	Stress study	*P* value
*General*			
Age, years	29 ± 7		
Gender, male/female	4/2		
*Metabolic parameters*			
BMI, Kg/m^2^	23 ± 4	24 ± 5	0.5
HbA1c, mmol/mol	34.0 ± 1.5	32.0 ± 2.2	0.2
Fasting glucose, mmol/L	5.1 ± 0.3	5.2 ± 0.3	0.6
Glucose 1 h post OGTT	6.6 ± 1.2	6.6 ± 1.2	1
Resting systolic BP, mmHg	122 ± 5	124 ± 9	0.6
Resting HR, bpm	68 ± 9	65 ± 13	0.5
*Echocardiography*			
E/A ratio	1.5 ± 0.6		
E/e’, mean	6.0 ± 1.1		
*CMR*			
LVEF, % (range)	55 ± 4 (51–61)	56 ± 3 (51–60)	0.8
LVEDV index, ml/m^2^	76 ± 13	72 ± 11	0.2
LVESV index, ml/m^2^	33 ± 3	29 ± 7	0.4
LV mass index, g/m^2^	55 ± 2	58 ± 3	0.1

BMI, body mass index; BP, blood pressure; HbA1C, glycated haemoglobin; HR, heart rate; E/A, early to late peak diastolic transmitral flow velocity ratio; E/e', early diastolic transmitral flow velocity to early diastolic mitral annular tissue velocity ratio; CMR, cardiovascular magnetic resonance; LVEF, left ventricular ejection fraction; LVEDV, left ventricular end-diastolic volume; LV mass, left ventricular mass

*Significance P < 0.05

**Table 2:** Haemodynamic response during adenosine infusion (mean ± SD)

	Before	During	*P* value
HR, bpm	65 ± 13	108 ± 11	< 0.001
Rate-pressure product, mmHg*HR/10^3^	8.2 ± 1.1	12.3 ± 1.2	0.03
LVEDV, ml	138 ± 29	121 ± 24	0.04
LVESV, ml	62 ± 15	52 ± 15	0.2
SV, ml	77 ± 15	68 ± 15	0.09

HR, heart rate; LVEDV, left ventricular end-diastolic volume; LVESV, left ventricular end-systolic volume; SV, stroke volume

### Safety data regarding hyperpolarized [1-^13^C]pyruvate injection

Participants received a total of two successful injections of [1-^13^C]pyruvate. The mean level of polarization was 25 ± 9% and pH was 7.7 ± 0.1. Time from dissolution to injection was < 150 s. No adverse events occurred.

### Hyperpolarized [1-^13^C]pyruvate CMR imaging

**Fig. 3:**
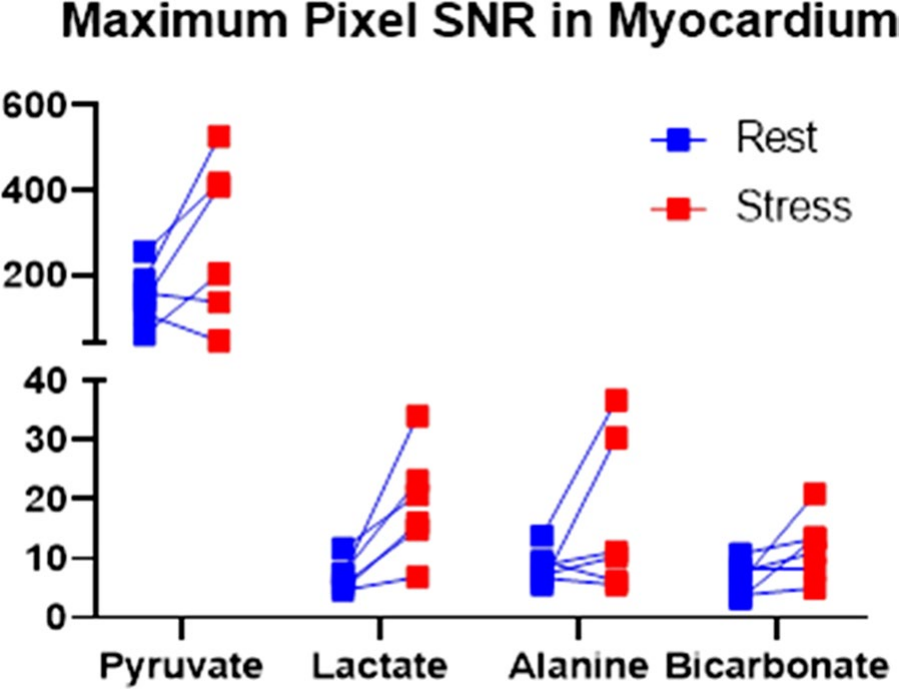
Signal to noise ratio. The blue squares represent signal-to-noise ratio (SNR) at rest and the red squares represent SNR during stress

**Fig. 4:**
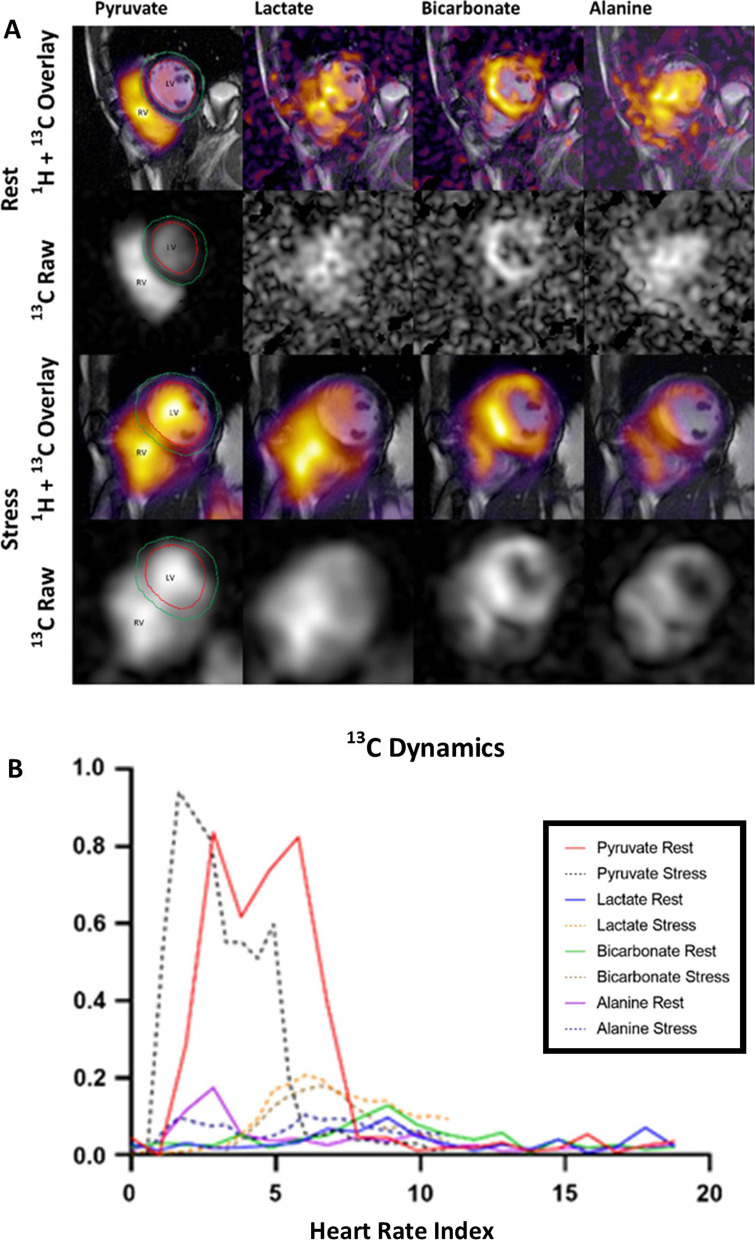
**A** Example of visualization of [1-^13^C]pyruvate and its downstream metabolites from arrival of pyruvate in the lumen of the right ventricle (RV) and left ventricle (LV) to downstream appearance of [1-^13^C]lactate, [^13^C]bicarbonate and [1-^13^C]alanine. Metabolite data are shown overlaid an anatomical cine image and as raw metabolite images. Region of interest is the myocardium of the LV. The red line delineates the endocardium and the green line delineates the epicardium. **B** An example of temporal dynamics for [1-^13^C]pyruvate and metabolites is shown. As hyperpolarized data were acquired per heart cycle, we have shown the data indexed to heart rate at rest and during stress to depict how heart rate changes the temporal dynamics

The obtained data of hyperpolarized [1-^13^C]pyruvate had a signal-to-noise ratio (SNR) of 151 ± 67 at rest and 289 ± 189 during stress. The metabolite SNR were increased from rest to stress: 7 ± 3 to 19 ± 9 for [1-^13^C]lactate, 7 ± 3 to 12 ± 5 for [^13^C]bicarbonate and 9 ± 3 to 17 ± 13 for [1-^13^C]alanine (Fig. 4). The time-to-peak (TTP) for metabolites increased from rest to stress: 13 ± 4 s to 16 ± 7 s (*p* = 0.3) for [1-^13^C]lactate, 13 ± 4 s to 17 ± 7 s for [^13^C] (*p* = 0.1) and 6 ± 3 to 11 ± 9 s (*p* = 0.3) for [1-^13^C]alanine. An example of HP [1-^13^C]pyruvate CMR imaging and temporal dynamics are shown in Fig. 3.

**Fig. 5:**
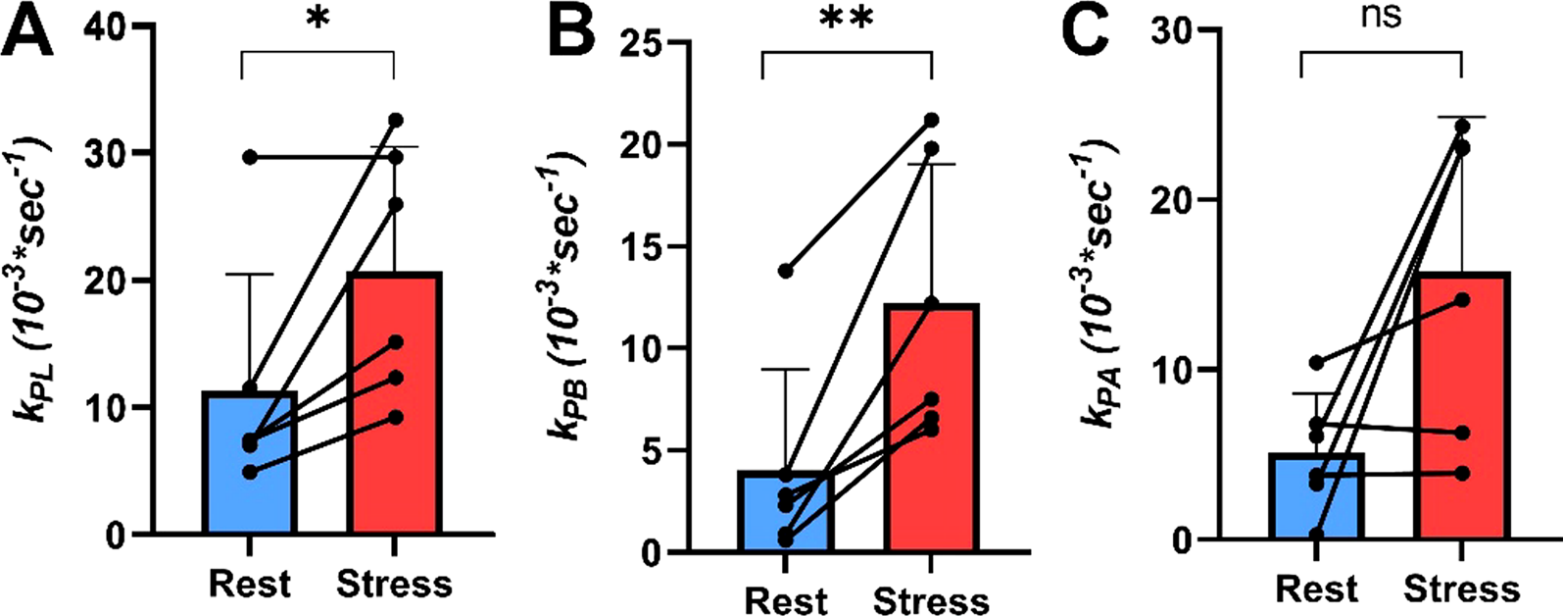
Conversion rate constants. **A**–**C** The mean *k*_PL,_ *k*_PB_ and *k*_PA_ at rest and during stress. The mean *k*_PL_ increased statistically significantly from 11 ± 9 *10^–3^ s^−1^ to 20 ± 10 *10^–3^ s^−1^, *p* = 0.04. The mean *k*_PB_ increased statistically significantly from 4 ± 4 *10^–3^ s^−1^ to 12 ± 7 *10^–3^ s^−1^, *p* = 0.008. The *k*_PA_ increased from 5 ± 3 *10^–3^ s^−1^ to 16 ± 9 *10^–3^ s^−1^, *p* = 0.06

**Fig. 6:**
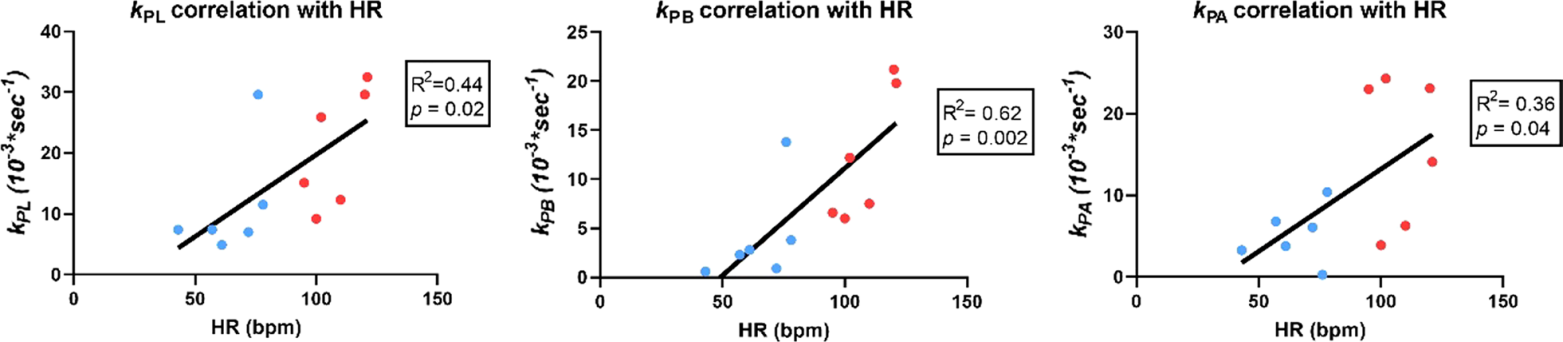
Conversion rate constants correlation with HR. There was a positive and statistically significant correlation of HR and increase in *k*_P_ for both lactate (*p* = 0.02), bicarbonate (*p* = 0.002) and alanine (*p* = 0.04). HR, heart rate

TTP perfusion and first order moment (FM) perfusion of [1-^13^C]pyruvate were assessed in the myocardium at the mid-LV level. TTP was significantly reduced from 6.2 ± 2.8 to 2.7 ± 1.3 s, *p* = 0.02, and FM was reduced from 17.8 ± 5.5 to 7.1 ± 2.0 s, *p* = 0.005. The *k*_PL_ increased from 11 ± 9 *10^–3^ s^−1^ to 20 ± 10 *10^–3^ s^−1^, *p* = 0.04. The *k*_PB_ increased from 4 ± 4 *10^–3^ s^−1^ to 12 ± 7 *10^–3^ s^−1^, *p* = 0.008. Finally, the *k*_PA_ increased from 5 ± 3 *10^–3^ s^−1^ to 16 ± 9 *10^–3^ s^−1^, *p* = 0.06 (Figs. 4, 5). We found a positive and significant correlation of HR and increase in *k*_PL_ (*p* = 0.02), *k*_PB_ (*p* = 0.002) and *k*_PA_ (*p* = 0.04) (Fig. 6).

## Discussion

The present study is the first-in man demonstration of the physiological changes in lactate dehydrogenase and PDH flux in response to adenosine stress in the healthy human heart, using HP [1-^13^C]pyruvate CMR. We document that HP [1-^13^C]pyruvate CMR in combination with a standard adenosine stress protocol was well tolerated in healthy human subjects. We demonstrated that increased HR was associated with an increased metabolism of [1-^13^C]pyruvate in the normal myocardium. Our findings suggest a faster pyruvate oxidation due to PDH activation in response to an increased myocardial workload.

### Metabolism in the healthy human heart

Participants were fasted for 8 to 15 h and oral glucose loading was used to maximize PDH flux and standardize metabolic conditions in both the rest and stress study [16, 20, 21]. Adenosine is a validated and widely used pharmacological stress test in cardiac imaging [10, 22, 23]. We found significant increases in HR, rate pressure product and cardiac output during the adenosine stress. The HP observations and the haemodynamic response must reflect both the systemic and coronary effects of adenosine. Adenosine induces coronary and systemic vasodilatation and reflex tachycardia [24]. Semi-parametric measures of the large blood pool component of [1-^13^C]pyruvate was used to evaluate the hemodynamic response to adenosine. We found a significant reduction in the TTP and FM for [1-^13^C]pyruvate in the mid-LV myocardium (inversely correlated to perfusion [25]) consistent with increased HR and thus pyruvate delivery. The increased myocardial signal of pyruvate during stress, suggest an enhanced myocardial [1-^13^C]pyruvate uptake likely due to coronary vasodilation, consistent with previous experimental findings [26]. However, unfortunately we cannot distinguish how much of the increased [1-^13^C]pyruvate signal is derived from increased myocardial uptake and how much is caused by increased vascular signal due to vasodilation. In this regard, it is interesting that we observed a tendency towards increased [1-^13^C]alanine signal which has been suggested as a surrogate measure for pyruvate uptake [16]. We observed a statistically significant increases in PDH flux as well as [1-13C]pyruvate to [1-^13^C] lactate exchange during stress. The relative increase in [1-^13^C]lactate signal is explained by [1-^13^C]pyruvate to [1-^13^C]lactate exchange, which depend on the delivery of pyruvate, on the rate of pyruvate uptake and subsequently on the concentration of LDH and its substrate pool sizes. The relative increase in PDH flux was larger, demonstrating that the healthy heart can increase oxidative energy production during moderate stress. This agrees with previous animal studies and invasive human studies, showing activation of PDH and increased pyruvate oxidation in response cardiac stress [27]. Interestingly, a similar metabolite dynamic pattern (TTP) of the individual metabolites was observed, which seem to contradict the increased metabolic conversion (TTP of the individual metabolite dynamic curve is inversely correlated with the apparent rate constant of the individual metabolites) [28]. It is important to note that the temporal dynamics of the metabolites are 3 times lower than that of pyruvate and thus, could at least partly explain this surprising finding. Also, given the voxel size, partial volume effects may influence these results. Finally, due to coronary vasodilation, coronary vasculature may be a significant part of the pyruvate signal in the myocardium and thus may influence TTP. Further studies are needed to solve these issues.

### Feasibility and tolerability of rest-stress HP [1-^13^C]pyruvate CMR imaging

In line with previous studies [10–14], we found that all participants tolerated HP [1-^13^C]pyruvate well and all participants returned for the second examination. Furthermore, we found no adverse reactions when HP [1-^13^C]pyruvate infusion was combined with adenosine infusion.

## Limitations

Several limitations should be considered in relation to the dDNP method and the present study. First, our sample size was small and the method is currently limited to only 24 centers around the world. The method is currently technically demanding and requires dedicated hardware and trained staff to produce HP probes and interpret data. However, the development of the commercially available, clinical 5 T SPINlab (General Electric Healthcare) and automation of several technical steps have improved the workflow of HP CMR significantly, decreasing the barrier for clinical adaptation [29, 30]. Second, the rest and stress data were collected on separate days. However, resting HR, blood pressure and fasting blood glucose were similar on the two study days, indicating similar metabolic states. Third, a limitation to the perfusion measurements is that pyruvate is metabolised which could bias the peak signal. Future studies, using a metabolically inactive probe such as ^13^C urea could be used to address this issue [31]. Forth, the high flip angles used to image metabolites increase the impact of imperfect slice profiles. However, the kinetic model applied in the present study does not require perfect saturation, albeit the RF inhomogeneity would impact the absolute rate constant estimations. We used each subject as its own comparison, and as B1 calibration was kept constant between the two examinations in the same individual, we do not believe that the 90° degree pulses extensively affect our conclusions. Finally, we did not do a sham adenosine infusion. One could argue that the infused volume of adenosine itself, could influence cardiac work and metabolism. However, as the infused volume was 60–70 mL, we believe this effect to be negligible. Future studies on patients with CAD and chronic HF are needed to evaluate the true clinical implications of HP [1-^13^C]pyruvate CMR in combination with adenosine stress test. In addition, a protocol using the ß-adrenergic agent dobutamine [23] would also be an important topic for future research.

## Conclusions

The present study represents the first-in-human non-invasive, real-time, *in-vivo* investigation of adenosine stress-induced metabolic changes in the healthy human heart using HP [1-^13^C]pyruvate CMR. The study confirms that it is feasible and well tolerated to add an adenosine stress test to HP [1-^13^C]pyruvate CMR. In addition, the study demonstrates an increased pyruvate oxidation during low to moderate cardiac stress. Finally, this study forms the basis for comparisons in studies of cardiac diseases.

## Data Availability

The datasets used and analysed during the current study are available from the corresponding author on reasonable request.

## Abbreviations

BMI: Body mass index
bSSFP: Balanced steady-state free precession
CAD: Coronary artery disease
CMR: Cardiovascular magnetic resonance
dDNP: Dissolution dynamic nuclear polarization technique
ECG: Electrocardiogram
FM: First order moment
HF: Heart failure
HP: Hyperpolarized
HR: Heart rate
k_pv_: Pyruvate oxidation rate
k_pl_: Conversion rate constant
LDH: Lactate dehydrogenase
LV: Left ventricle/left ventricular
LVEF: Left ventricular ejection fraction
PDH: Pyruvate dehydrogenase
RF: Radiofrequency
SNR: Signal-to-noise ratio
SPSP: Spectral special
SVD: Singular volue decomposition
TTP: Time to peak

## References

[1] Knuuti J, Wijns W, Achenbach S, Agewall S, Barbato E, Bax JJ (2020). 2019 ESC guidelines for the diagnosis and management of chronic coronary syndromes. Eur Heart J.

[2] Cleland JGF, Calvert M, Freemantle N, Arrow Y, Ball SG, Bonser RS (2011). The heart failure revascularisation trial (HEART). Eur J Heart Fail.

[3] Hassanabad AF, MacQueen KT, Ali I (2019). Surgical Treatment for Ischemic Heart Failure (STICH) trial: A review of outcomes. J Card Surg.

[4] Ardenkjaer-Larsen JH (2016). On the present and future of dissolution-DNP. J Magn Reson.

[5] Cunningham CH, Lau JYC, Chen AP, Geraghty BJ, Perks WJ, Roifman I (2016). Hyperpolarized 13C Metabolic MRI of the Human Heart: Initial Experience. Circ Res.

[6] Rider OJ, Apps A, Miller JJ, Lau JYC, Lewis AJM, Peterzan MA (2020). Noninvasive in vivo assessment of cardiac metabolism in the healthy and diabetic human heart using hyperpolarized 13C MRI. Circ Res.

[7] Park JM, Reed GD, Liticker J, Putnam WC, Chandra A, Yaros K (2020). Effect of Doxorubicin on Myocardial Bicarbonate Production from Pyruvate Dehydrogenase in Women with Breast Cancer. Circ Res.

[8] Apps A, Lau J, Peterzan M, Neubauer S, Tyler D, Rider O (2018). Hyperpolarised magnetic resonance for in vivo real-time metabolic imaging. Heart.

[9] Apps A, Lau JYC, Miller JJJJ, Tyler A, Young LAJ, Lewis AJM, et al. Proof-of-Principle Demonstration of Direct Metabolic Imaging Following Myocardial Infarction Using Hyperpolarized 13C CMR. Vol. 14, JACC: Cardiovascular Imaging. Elsevier Inc.; 2021. p. 1285–8. 10.1016/j.jcmg.2020.12.023 PMC8184499 33582059

[10] Kitkungvan D, Lai D, Zhu H, Roby AE, Johnson NP, Steptoe DD (2017). Optimal adenosine stress for maximum stress perfusion, coronary flow reserve, and pixel distribution of coronary flow capacity by kolmogorov-smirnov analysis. Circ Cardiovasc Imaging.

[11] Zhu Z, Zhu X, Ohliger MA, Tang S, Cao P, Carvajal L (2019). Coil combination methods for multi-channel hyperpolarized 13 C imaging data from human studies. Circ.

[12] Schulte RF, Sacolick L, Deppe MH, Janich MA, Schwaiger M, Wild JM (2011). Transmit gain calibration for nonproton MR using the Bloch-Siegert shift. NMR Biomed.

[13] Grist JT, Sánchez-heredia ES, Mclean MA, Tougaard R, Riemer F, Schulte RF (2020). Creating a clinical platform for carbon-13 studies using the sodium-23 and proton resonances. Magn Reson Imaging.

[14] Schulte RF, Sperl JI, Weidl E, Menzel MI, Janich MA, Khegai O (2013). Saturation-recovery metabolic-exchange rate imaging with hyperpolarized [1–13C] pyruvate using spectral-spatial excitation. Magn Reson Med.

[15] Schulte RF, Wiesinger F (2013). Direct design of 2D RF pulses using matrix inversion. J Magn Reson.

[16] Tougaard RS, Szocska Hansen ES, Laustsen C, Nørlinger TS, Mikkelsen E, Lindhardt J (2019). Hyperpolarized [1- 13 C]pyruvate MRI can image the metabolic shift in cardiac metabolism between the fasted and fed state in a porcine model. Magn Reson Med.

[17] Larson PEZ, Gordon JW (2021). Hyperpolarized metabolic mri—acquisition, reconstruction, and analysis methods. Metabolites.

[18] Chen HY, Aggarwal R, Bok RA, Ohliger MA, Zhu Z, Lee P (2020). Hyperpolarized 13C-pyruvate MRI detects real-time metabolic flux in prostate cancer metastases to bone and liver: a clinical feasibility study. Prostate Cancer Prostatic Dis.

[19] Autry AW, Gordon JW, Chen HY, LaFontaine M, Bok R, Van Criekinge M (2020). Characterization of serial hyperpolarized 13C metabolic imaging in patients with glioma. NeuroImage Clin..

[20] Rider OJ, Apps A, Miller JJJJ, Lau JYC, Lewis AJM, Peterzan MA (2020). Noninvasive in vivo assessment of cardiac metabolism in the healthy and diabetic human heart using hyperpolarized 13C MRI. Circ Res.

[21] Timm KN, Apps A, Miller JJ, Ball V, Chong CR, Dodd MS (2018). Assessing the optimal preparation strategy to minimize the variability of cardiac pyruvate dehydrogenase flux measurements with hyperpolarized MRS. NMR Biomed.

[22] Vincenti G, Masci PG, Monney P, Rutz T, Hugelshofer S, Gaxherri M (2017). Stress Perfusion CMR in Patients With Known and Suspected CAD: prognostic value and optimal ischemic threshold for revascularization. JACC Cardiovasc Imaging.

[23] Paetsch I, Jahnke C, Wahl A, Gebker R, Neuss M, Fleck E (2004). Comparison of dobutamine stress magnetic resonance, adenosine stress magnetic resonance, and adenosine stress magnetic resonance perfusion. Circulation.

[24] Bush A, Busst CM, Clarke B, Barnes PJ (1989). Effect of infused adenosine on cardiac output and systemic resistance in normal subjects. J Clin Pharmac.

[25] Carlsson M, Wilson M, Martin A, Saeed M (2009). Myocardial microinfarction aF Ter coronary microembolization in swine: MR imaging characterization. Radiology.

[26] Lau AZ, Miller JJ, Robson MD, Tyler DJ (2017). Simultaneous assessment of cardiac metabolism and perfusion using copolarized [1-13C]pyruvate and 13C-urea. Magn Reson Med.

[27] Stanley WC, Recchia FA, Lopaschuk GD (2005). Myocardial substrate metabolism in the normal and failing heart. Physiol Rev.

[28] Daniels CJ, Mclean MA, Schulte RF, Robb FJ, Gill AB, Mcglashan N (2016). A comparison of quantitative methods for clinical imaging with hyperpolarized 13C-pyruvate. NMR Biomed.

[29] Wang ZJ, Ohliger MA, Larson PEZ, Gordon JW, Bok RA, Slater J (2019). Hyperpolarized 13C MRI: State of the art and future directions. Radiology.

[30] Jørgensen SH, Bøgh N, Hansen ESS, Væggemose M, Wiggers H, Laustsen C (2021). Hyperpolarized MRI – An update and future perspectives. Semin Nucl Med.

[31] Qin H, Tang S, Riselli AM, Bok RA, Delos Santos R, van Criekinge M (2022). Clinical translation of hyperpolarized 13C pyruvate and urea MRI for simultaneous metabolic and perfusion imaging. Magn Reson Med.

